# Stromal Cell-Derived Factor 1 Regulates the Actin Organization of Chondrocytes and Chondrocyte Hypertrophy

**DOI:** 10.1371/journal.pone.0037163

**Published:** 2012-05-18

**Authors:** Koichi Murata, Toshiyuki Kitaori, Shinya Oishi, Naoki Watanabe, Hiroyuki Yoshitomi, Shimei Tanida, Masahiro Ishikawa, Takashi Kasahara, Hideyuki Shibuya, Nobutaka Fujii, Takashi Nagasawa, Takashi Nakamura, Hiromu Ito

**Affiliations:** 1 Department of Orthopaedic Surgery, Kyoto University Graduate School of Medicine, Sakyo, Kyoto, Japan; 2 Department of Chemogenomics, Kyoto University Graduate School of Pharmaceutical Sciences, Kyoto, Japan; 3 Laboratory of Single-Molecule Cell Biology, Tohoku University Graduate School of Life Science, Aoba, Sendai, Japan; 4 Department of Immunobiology and Hematology, Kyoto University, Institute for Frontier Medical Sciences, Kyoto, Japan; 5 Department of the Control for Rheumatic Diseases, Kyoto University Graduate School of Medicine, Kyoto, Japan; University of Nebraska Medical Center, United States of America

## Abstract

Stromal cell-derived factor 1 (SDF-1/CXCL12/PBSF) plays important roles in the biological and physiological functions of haematopoietic and mesenchymal stem cells. This chemokine regulates the formation of multiple organ systems during embryogenesis. However, its roles in skeletal development remain unclear. Here we investigated the roles of SDF-1 in chondrocyte differentiation. We demonstrated that SDF-1 protein was expressed at pre-hypertrophic and hypertrophic chondrocytes in the newly formed endochondral callus of rib fracture as well as in the growth plate of normal mouse tibia by immunohistochemical analysis. Using SDF-1^−/−^ mouse embryo, we histologically showed that the total length of the whole humeri of SDF-1^−/−^ mice was significantly shorter than that of wild-type mice, which was contributed mainly by shorter hypertrophic and calcified zones in SDF-1^−/−^ mice. Actin cytoskeleton of hypertrophic chondrocytes in SDF-1^−/−^ mouse humeri showed less F-actin and rounder shape than that of wild-type mice. Primary chondrocytes from SDF-1^−/−^ mice showed the enhanced formation of philopodia and loss of F-actin. The administration of SDF-1 to primary chondrocytes of wild-type mice and SDF-1^−/−^ mice promoted the formation of actin stress fibers. Organ culture of embryonic metatarsals from SDF-1^−/−^ mice showed the growth delay, which was recovered by an exogenous administration of SDF-1. mRNA expression of type X collagen in metatarsals and in primary chondrocytes of SDF-1^−/−^ mouse embryo was down-regulated while the administration of SDF-1 to metatarsals recovered. These data suggests that SDF-1 regulates the actin organization and stimulates bone growth by mediating chondrocyte hypertrophy.

## Introduction

Endochondral ossification is an essential process for skeletal development [1]. During skeletogenesis, mesenchymal cells aggregate at the sites where skeletal elements will eventually be formed, and they differentiate into chondrocytic lineage and proliferate. The cartilage templates are composed of nonhypertrophic (resting and proliferating) and hypertrophic chondrocytes as well as vast amounts of extracellular matrix produced by chondrocytes [1]. Chondrocyte hypertrophy is a unique step in chondrocyte differentiation, in which the cells become bigger and rather transparent, and is a mandatory event allowing calcification, vascularization, osteoblast differentiation, and remodeling into bone to occur [2].

A number of secreted or intracellular polypeptides cooperatively regulate the transition of chondrocyte hypertrophy. PTH-related peptide, Indian hedgehog, fibroblast growth factors, SRY (sex determining region Y)-box 9 (Sox9) and Wnt proteins are reportedly essential factors for chondrocyte hypertrophy [3]–[8]. However, much less is known about the intracellular events, such as cytoskeletal reorganization that can allow the cell size and shape to change.

Stromal cell-derived factor-1 (SDF-1)/pre-B cell growth-stimulating factor belongs to CXC subfamily of chemokines as CXCL12 [9], [10]. SDF-1 signals through a G-protein-coupled receptor, C-X-C chemokine receptor 4 (CXCR4) [10], [11] and, CXCR7 [12], [13], and critical roles of SDF-1/CXCR4 in hematopoietic stem cells (HSCs) have been extensively reported.

During the last decade, accumulating data have supported an emerging hypothesis that SDF-1/CXCR4 also plays pivotal roles in the biological and physiological functions of mesenchymal stem cells (MSCs) [14], [15]. SDF-1 is up-regulated at sites of injuries and serves as a potent chemoattractant to recruit circulating or residing CXCR4-expressing MSCs which are necessary for a tissue-specific organ repair or regeneration in liver [16], heart [17], [18], brain [19], kidney [20], and skin [21]. We have recently demonstrated that SDF-1 recruited MSCs to bone repairing sites in the acute phase of endochondral bone repair [22]. However, little is known on roles of SDF-1/CXCR4 signal on the bone growth and the endochondral bone formation.

In the process of chondrogenesis, chondrocytes change cell shape from a fibroblastoid to a round or polygonal morphology, which is called as hypertrophic conversion [23], a unique and crucial step in chondrocyte differentiation. The molecular mechanisms responsible for this cell shape change are largely unknown, but the actin cytoskeleton presumably plays important roles in this context. The only intracellular signaling known to associate with the actin reorganization during chondrocyte differentiation is RhoA/ROCK [24]–[26]. The reports on RhoA/ROCK suggest that the changes in morphology have important roles in chondrocyte differentiation. On the other hand, SDF-1 induces cytoskeleton rearrangements in homing and migration of hematopoietic cells through Phosphoinositide 3-kinase/Akt and RhoA/ROCK signaling [27], [28], which can lead to an assumption of the important roles of SDF-1 on the shape and size changes of chondrocytes. This assumption, however, remains to be largely investigated.

We herein demonstrated that SDF-1 is crucial for endochondral bone development. The embryonic humeri of SDF-1^−/−^ mice were shorter than those of wild-type mice, especially prominent in the hypertrophic zone. The actin cytoskeleton of SDF-1^−/−^ chondrocytes in the humeri and in monolayer culture was disturbed. With cultured metatarsal explants of SDF-1^−/−^mice, the lack of SDF-1 impaired the development of metatarsals and chondrocyte hypertrophy, and the addition of SDF-1 reversed the impairments. Our results strongly suggest that SDF-1 regulates actin polymerization and stimulates bone growth by mediating chondrocyte hypertrophy.

## Results

### Distribution of SDF-1 in the Growth Plate and the Endochondral Callus

**Figure 1:**
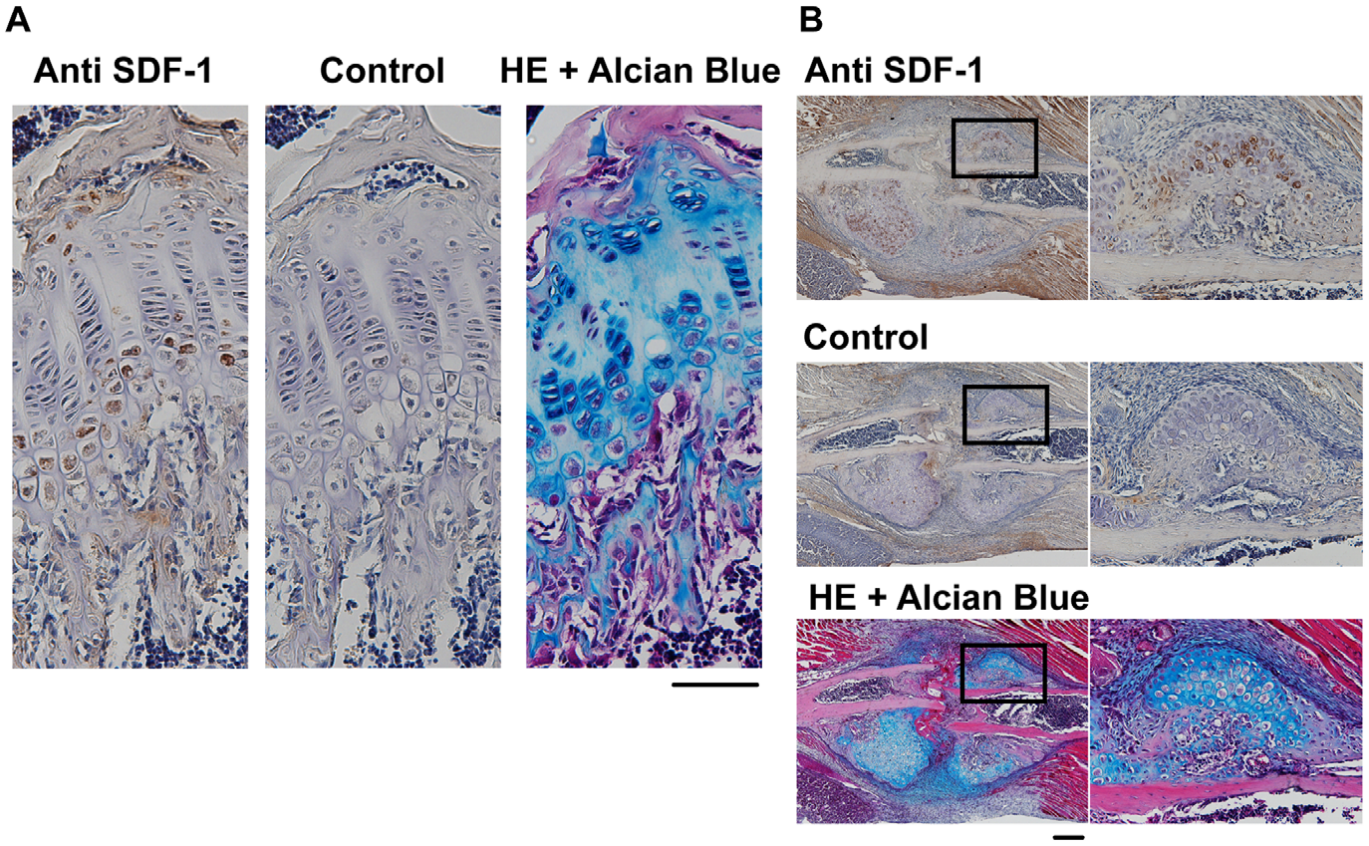
SDF-1 protein was expressed at the prehypertrophic and hypertrophic zones. The growth plate of 4-week-old mouse tibia (A) and endochondral callus of rib fracture (B) were stained with hematoxylin and eosin (HE), or immunohistochemically stained with antiSDF-1 antibody or IgG (control). Boxed areas in the panel are shown in a higher magnification (×200) in the right panels. The result is representative of three separate experiments; Scale bar, 200 µm.

To evaluate potential roles of SDF-1 in endochondral bone formation, we analyzed the distribution of SDF-1 in the tibial growth plates from 4-week-old wild-type mice and newly formed endochondral callus of rib fracture model at day 10 by immunohistochemistry. SDF-1 was expressed at prehypertrophic and hypertrophic chondrocytes both in the growth plate (**Figure 1A**) and in the endochondral callus (**Figure 1B**), suggesting important roles of SDF-1 on the transition from prehypertrophic to hypertrophic chondrocytes in endochondral bone formation.

### Delayed Growth and Reduced Size of the Hypertrophic Zone of SDF-1^−/−^ Mouse Humeri

**Figure 2:**
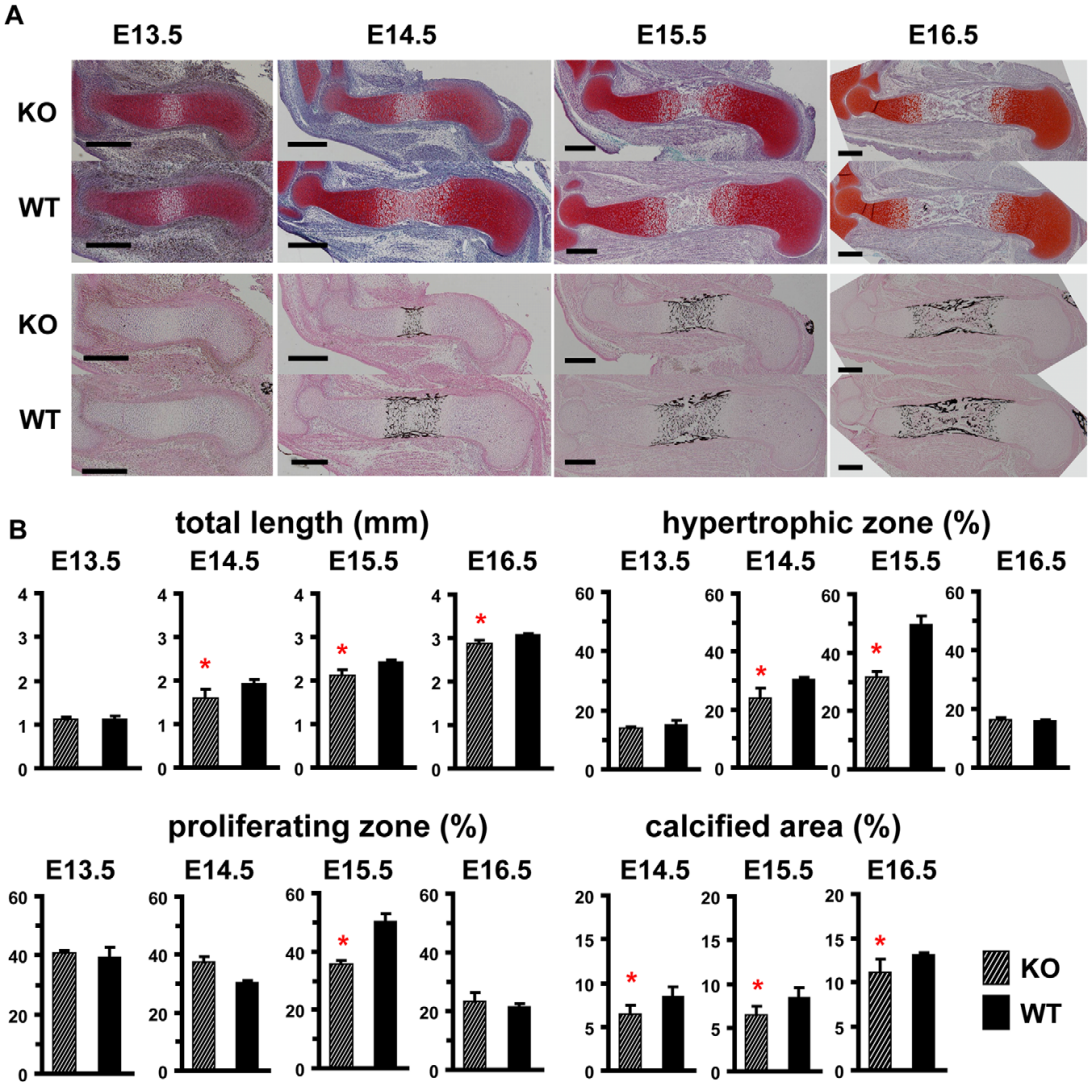
Growth of the embryonic humeri of SDF-1^−/−^ mice delays. **A**: The embryonic humeri of wild-type (WT) and SDF-1^−/−^ (KO) mice. Specimens were processed to paraffin-embedded sections and stained with hematoxylin, eosin (lower panels) and alcian blue (upper panels). **B**: Length of the humeri. The total length of the humeri and the ratios of the proliferating, the hypertrophic, or the calcified zone in each day were calculated as described in Methods section. Values are the mean and standard deviation of more than three independent experiments; *, P<0.05; Scale bar, 300 µm.

To investigate the effect of SDF-1 in endochondral bone formation, we next evaluated the phenotypic differences between SDF-1^−/−^ and wild-type mice. The humeri were resected from embryos at E13.5, E14.5, E15.5, and E16.5 (n = 4 at each point), and processed in paraffin sections (**Figure 2A**). The lengths of the total humeri and the ratios of proliferating, hypertrophic, and calcified zones were measured (**Figure 2B**). The total length of the whole humerus of SDF-1^−/−^ mice was shorter than that of wild-type mice in E14.5, E15.5, and E16.5 by 17.9%, 24.7%, and 6.8%, respectively, with statistical significances. To examine which zone contributed to the total length difference, we evaluated the proliferating, hypertrophic, and calcified zones of the humeri as the percentages against the total length. The ratio of hypertrophic zone was significantly smaller in SDF-1^−/−^ mice than in wild-type mice at E14.5 and E15.5 by 21.2% and 31.0%, respectively. The ratio of calcified zone was also smaller in SDF-1^−/−^ mice than in wild-type mice at E14.5 and E15.5 by 45.0% and 36.0%, respectively, with statistical significances. The most marked difference was found at E15.5, especially in the hypertrophic zone, while no significant differences were observed in any zones at E13.5. No significant difference was found in cell proliferation rates between SDF-1^−/−^ and wild-type mice based on 5-Bromo-2′ deoxy-uridine (Brd-U) staining (**Figure** S**1**). These results demonstrated the absence of SDF-1 mainly affects the growth of the hypertrophic zone rather than the proliferating zone.

### Absence of SDF-1 Impairs Cytoskeleton of Hypertrophic Chondrocytes

**Figure 3:**
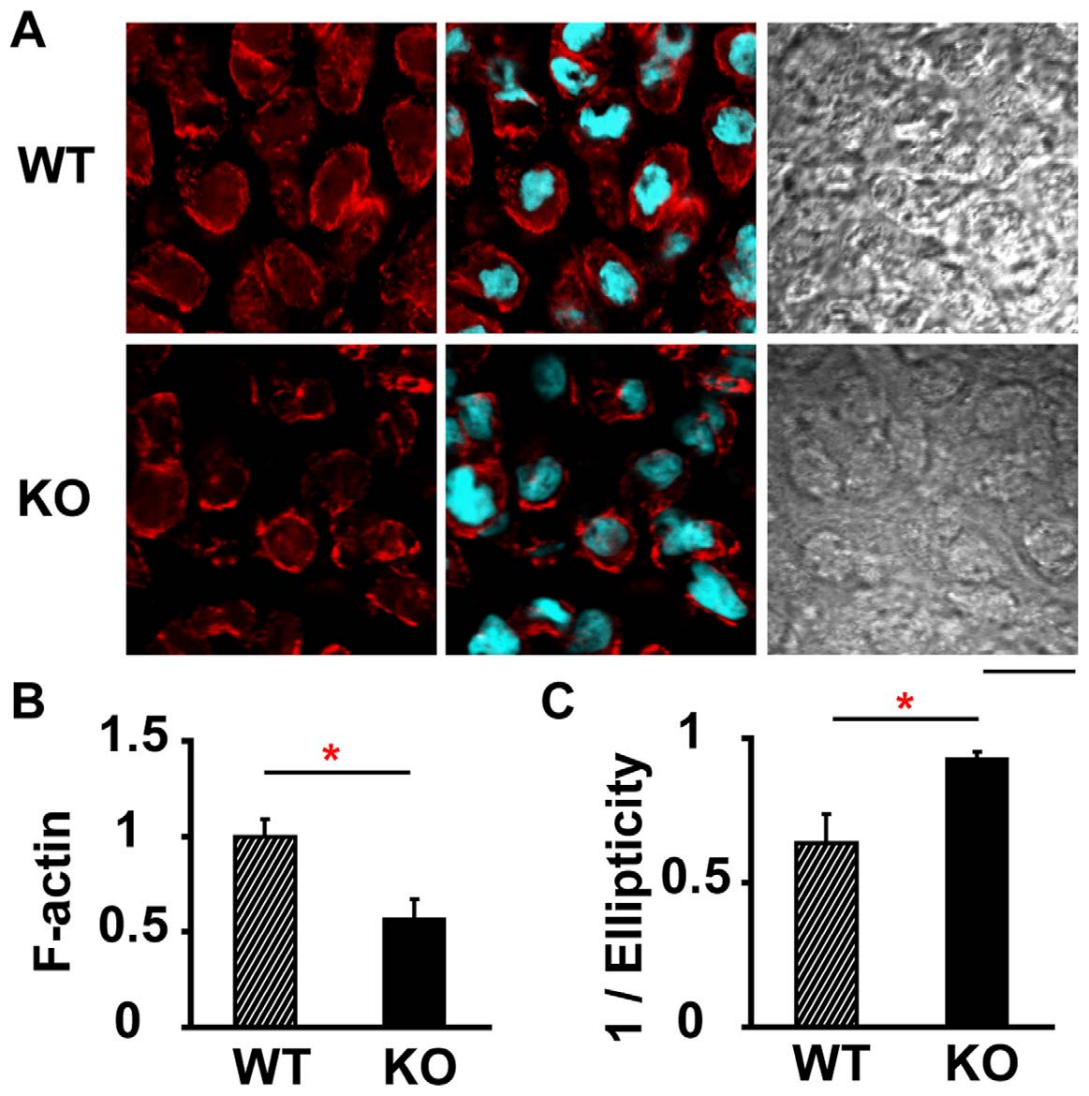
Cytoskeleton of hypertrophic chondrocytes in the humeri. **A**: Frozen sections of the humeri isolated from SDF-1^−/−^ (KO), and wild-type (WT) mice at E15.5, were fixed with paraformaldehyde and stained with Rhodamine-Phalloidin for actin filaments and SYBR Green for nuclei. Left panel, Rhodamine-Phalloidin; middle panel, merge of Rhodamine-Phalloidin and SYBR Green; right panel, differentiation interference image. Representative fields are shown. **B**: The intensity of actin filament staining (F-actin) was quantified with Image J software. Values were normalized to F-actin of WT chondrocytes. **C**: The ratios of the short axis to the long axis (1/Ellipticity) in chondrocytes were calculated with Image J. Values are the mean and s.e.m. of more than three separate experiments; *, P<0.05; Scale bar, 10 µm.

SDF-1 signaling leads to cytoskeleton rearrangements and integrin activation in hematopoietic cells [27]. Chondrocytes change their shape drastically from a fibroblastoid to a round shape during chondrogenesis. The reorganization of the actin cytoskeleton is also demonstrated to be important in hypertrophic transition of chondrocytes [24]. To determine whether SDF-1 affects the reorganization of the actin cytoskeleton and cellular morphology, we stained the frozen sections of the humeri from SDF-1^−/−^ and wild-type mouse embryos (E15.5) with Rhodamine-Phalloidin. No clear difference between SDF-1^−/−^ and wild-type hypertrophic chondrocytes was detected in the HE staining (data not shown) and in the differentiation interference image (**Figure 3A**). But interestingly, the Rhodamine-Phalloidin staining showed that the hypertrophic chondrocytes in SDF-1^−/−^ mice was rounder than wild-type mice. Quantification of F-actin revealed that F-actin content of hypertrophic chondrocytes in SDF-1^−/−^ mice was significantly lower than wild-type mice (P<0.05, **Figure 3B**). Next, to measure the ellipticity of chondrocytes, we calculated the ratio of a short axis to the long axis (1/ellipticity). The reciprocal of ellipticity was significantly higher in SDF-1^−/−^ chondrocytes than in wild-type chondrocytes (P<0.05, **Figure 3C**), which support the roundness of SDF-1^−/−^ hypertrophic chondrocytes. These tendencies were also observed in hypertrophic chondrocytes of SDF-1^−/−^ metatarsals (data not shown).

### SDF-1 Controls the Actin Organization of Primary Chondrocytes

**Figure 4:**
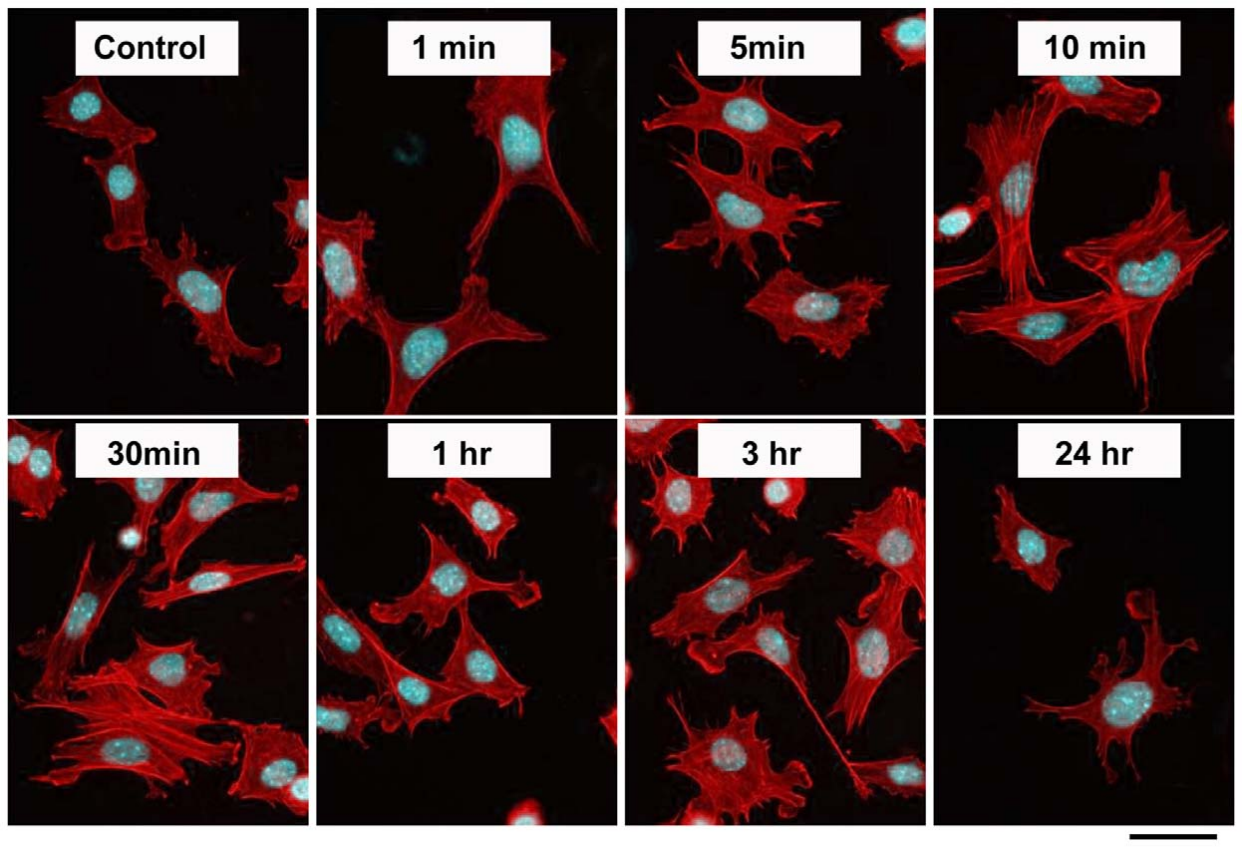
SDF-1 simulates actin assembly and stress fiber formation in a time-dependent manner. Primary chondrocytes from wild-type mice were treated with SDF-1 (100 ng/ml) as indicated. Cells were then fixed with paraformaldehyde and stained with Rhodamine-Phalloidin for actin filaments and 4′,6-diamidino-2-phenylindole for nuclei; Scale bar, 20 µm.

Based on the results described above, we investigated whether SDF-1 regulates the actin cytoskeleton of the chondrocytes, F-actin of primary chondrocytes were stained with Rhodamine-Phalloidin. Untreated chondrocytes from wild-type mice had a defined cortical rim, minimal stress fibers, and a polyhedral shape (**Figure 4**). With treatment of SDF-1 (100 ng/ml), the chondrocytes changed their shape into contractile rounded shape in a time-dependent manner. Incubation of the chondrocytes for 10 m with SDF-1 showed the actin stress fiber formation.

**Figure 5:**
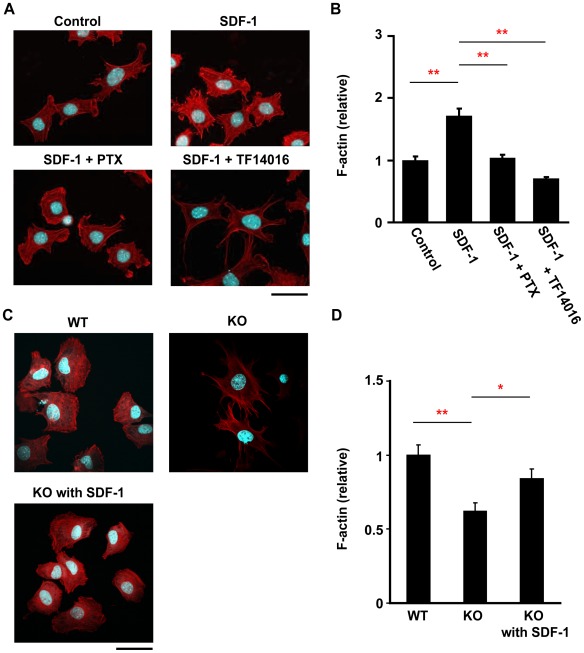
SDF-1/CXCR4 pathway controls actin cytoskeleton. **A**: Treatment with SDF-1 increased the actin filament density in primary chondrocytes. Primary chondrocytes from wild-type mice were treated with SDF-1 (100 ng/ml), or SDF-1 and CXCR4 specific antagonist, TF14016 (100 µM) or pertussis toxin (PTX, 100 ng/ml) for 60 m and stained with Rhodamine-Phalloidin. **B**: The F-actin content in chondrocytes was analyzed with Image J software. All values were normalized to F-actin of chondrocytes treated with conditioned medium only. **C**: Actin cytoskeleton of primary chondrocyte from SDF-1^−/−^ (KO), and wild-type (WT) mice. **D**: The F-actin content in chondrocytes was quantified with Image J software. All values were normalized to the F-actin of wild-type chondrocyte. Values are the mean and s.e.m. of more than three separate experiments; *, P<0.05, **,P<0.05; Scale bar, 20 µm.

Next, wild-type primary chondrocytes were treated with SDF-1, or SDF-1 and CXCR4 specific antagonist, TF14016 [29] (100 µM), or pertussis toxin (PTX, 100 ng/ml) for 60 m (**Figure** **5A**). Treatment with SDF-1 to primary chondrocytes resulted in contractile rounded cells with thick cortical rim of actin filaments. The chondrocytes treated with SDF-1 and TF14016 showed the similar morphology to untreated chondrocytes. Cells treated with SDF-1 and PTX showed slight induction of actin stress fibers. In quantitation of the F-actin in the Rhodamine-Phalloidin-stained cells, the F-actin content treated with SDF-1 for 60 m was significantly higher than the actin density of the chondrocytes treated with either medium alone, SDF-1+PTX, or SDF-1+TF14016 (**Figure 5B**).

Then we investigated the actin filaments of primary chondrocytes from SDF-1^−/−^ mice. Those chondrocytes clearly showed the enhanced formation of philopodia and the lost of F-actin through 7-day culture, compared with those of wild-type mice. The administration of recombinant SDF-1 to the chondrocytes of SDF-1^−/−^ mice inhibited the philopodia formation and the loss of F-actin. The cortical rim and shape of SDF-1^−/−^ chondrocytes with recombinant SDF-1 was similar to that of wild-type chondrocytes (**Figure 5C**). Quantification of F-actin revealed that actin density of SDF-1^−/−^ chondrocytes was lower than that of wild-type chondrocytes and of SDF-1^−/−^ chondrocytes incubated with recombinant SDF-1 (P<0.01, P<0.05, respectively) (**Figure 5D**). These results suggest that SDF-1/CXCR4 signaling strongly affects the reorganization of the actin cytoskeleton and the cellular morphology in primary chondrocytes.

### Deficit of SDF-1/CXCR4 Signaling Delays the Growth of Metatarsals in Organ Culture

**Figure 6:**
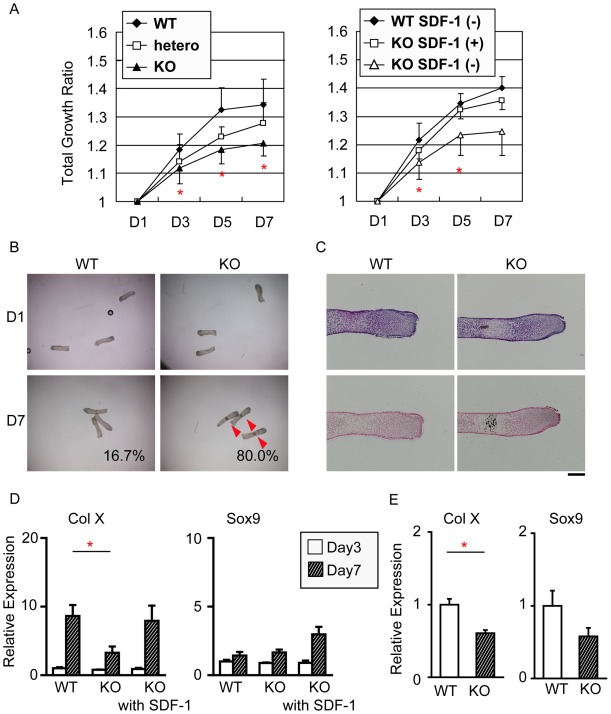
Lack of SDF-1 delays the growth metatarsals in organ culture. **A**: The metatarsal bones were harvested from SDF-1^−/−^ (KO), SDF-1+/− (hetero), and wild-type (WT) mice at E15.5, cultured for 7 days, and the total length was measured at day 1, 3, 5, and 7. The length of metatarsal bones was calculated by day 1 as controls. **B**: Calcified spots (arrows) were counted in KO and WT metatarsals at day 1 and 7. **C**: Metatarsal bones were stained with hematoxylin and eosin (upper panel), or von Kossa (lower panel); Scale bar, 200 µm. **D**: The expression levels of type X collagen (Col X) and Sox9 of cultured metatarsal bones from E15.5 WT and KO mice at day 3 and 10 were quantified by real-time PCR. **E**: Primary chondrocytes isolated from WT and KO mice were cultured for 3 weeks in chondrogenic medium supplemented with insulin. The expression levels of Col X and Sox9 were quantified by real-time PCR. Values are the mean and s.e.m. of more than three independent experiments; *, P<0.05.

The phenotypic changes of SDF-1^−/−^ mice exclusively occurred from E14.5 to E16.5. This period corresponds to the time of the vascular invasion, which indicates that molecular and/or cellular factors from circulation compensate for the lack of SDF-1. To verify the independent effects of endogenous SDF-1 in pre-vascularized bone, we used the primary metatarsal explant culture system. The metatarsal bones were harvested from wild-type mice at E15.5, cultured for 7 days, and the total length was measured at day 1, 3, 5 and 7 (**Figure 6**). We first confirmed that no vascular formation was observed in metatarsal bones at this stage by histology (data not shown).

As shown in **Figure 6A**, the metatarsal bones of wild-type mice kept up growing during the whole culture period and the ratio of the total length growth reached to 134% at day 7. The growth delayed at every time point in SDF-1+/− and SDF-1^−/−^ mice, and the ratios of the total length growth remained 128% and 121% at day 7, respectively. At the end of this culture assay, the total length of SDF-1^−/−^ metatarsal bones was 9.7% shorter than wild-type metatarsals with statistical significance, indicating the functional effect of endogenous SDF-1 in the normal bone growth. Then, to investigate whether SDF-1 can restore the perturbed growth of SDF-1^−/−^ metatarsal bones, 100 ng/ml of SDF-1 was added to the primary metatarsal culture system. The treatment of SDF-1 significantly regained the ratio of the total length growth of SDF-1^−/−^ metatarsals from 124% to 137% at day 7, which was the similar growth rate of wild-type metatarsals. Interestingly, during this assay, we observed calcified zones, verified by von Kossa staining, were found in 80.0%, 69.2%, and 16.7% of wild-type, SDF-1+/−, and SDF-1^−/−^ metatarsals, respectively at day 7 (**Figures 6B, C**), indicating that the delayed differentiation of hypertrophic chondrocytes affects the calcification of those cells in SDF-1^−/−^ mice, which was consistent with the phenotype of the SDF-1^−/−^ humerus (**Figure 2**).

These results strongly support the notion that self-produced SDF-1 is crucial for endochondral bone development at the pre-vascularized stage.

### SDF-1 Deficiency Impairs Type X Collagen Gene Expression

To examine the effect of SDF-1 to the phenotypical expression of type X collagen (Col X), and Sox9, metatarsal bones from E15.5 wild-type and SDF-1^−/−^ mouse embryos were cultured with or without SDF-1 for 3 or 7 days, and total RNAs were extracted and quantified by real-time PCR.

The expression of Col X mRNA increased from day 3 to day 7 in untreated wild-type metatarsals (**Figure 6D**), which demonstrated the normal growth of the explant culture. The expression of Col X mRNA decreased significantly at day 7 in untreated SDF-1^−/−^ metatarsals. The administration of recombinant SDF-1 to SDF-1^−/−^ metatarsals recovered the expression of Col X. There was no statistically significant difference in Sox9 expression.

Furthermore, primary chondrocytes isolated from wild-type and SDF-1^−/−^ mice were cultured for 3 weeks in chondrogenic medium supplemented with insulin to induce chondrocyte differentiation. Chondrocytes from SDF-1^−/−^ mice showed decreased Col X expression compared with that from wild-type mice (**Figure 6E**). The expression of Sox9 showed no statistically significant difference.

## Discussion

SDF-1/CXCR4 plays important roles in the biological and physiological functions of MSCs [14], [15], serving as a potent chemoattractant to recruit circulating or residing CXCR4-expressing MSCs to injured organs [16]–[21], including the fracture site of bones [22]. SDF-1 also plays crucial roles in the formation of multiple organ systems during embryogenesis [30]–[32], and it has been shown to associate with several diseases involving the skeleton, including rheumatoid arthritis and cancers that metastasize to the bone [33], [34]. In the present study we showed, for the first time, the promotion of chondrocyte hypertrophy with SDF-1 and contribution of SDF-1 to the actin reorganization, which had been shown to be important for chondrocyte differentiation.

During the endochondral bone development, SDF-1 is expressed at the growth plate, but the precise localization of SDF-1 in the growth plate is controversial. A previous study showed that SDF-1 was expressed in the central region of the proliferating zone of the growth plate at E14.5 and it was expressed in the periosteum and central region of the proliferating zone at E16.5 [35]. On the other hand, another study showed that SDF-1 was expressed in the area adjacent to the hypertrophic zone of the growth plate [36]. Immunohistochemical analysis of the current study revealed that SDF-1 protein was expressed in hypertrophic chondrocytes of the growth plates of the tibia and the fracture callus (**Figure 1**) as well as the hypertrophic zone of mouse embryos (data not shown). These data suggest that SDF-1 has important, physiological roles in the hypertrophic zone during the endochondral bone formation even after birth.

SDF-1 is involved in the migration of hematopoietic progenitors and morphogenesis of leukocytes [37]. In the processes, SDF-1 induces morphological changes in adherent leukocytes and the acquisition of a bipolar shape with front leading edges [38], [39], by stimulating the actin polymerization in a Rac and Cdc42-dependent fashion [38], and activating the small GTPases that control the actin cytoskeleton [40]. On the other hand, the actin cytoskeleton plays important roles in cellular homeostasis, proliferation and differentiation [41], [42]. Even in the hypertrophic transition during chondrocyte differentiation, chondrocytes change the cell shape from a fibroblastoid to a round or polygonal morphology [23] and, as an upstream regulator of cytoskeletal dynamics, RhoA/ROCK signaling was shown to associate with chondrocyte hypertrophy [43]. In this study, incubation of wild-type and SDF-1^−/−^ chondrocytes with SDF-1 changed the cell shape with an increase of F-actin density (**Figure 4**). In accordance with this fact, actin cytoskeletons of wild-type and SDF-1^−/−^ hypertrophic chondrocytes in the humeri were different in the amount and the shape (**Figure 3**). These results suggest that SDF-1 signaling affects the reorganization of the actin cytoskeleton and the maintenance of the cellular morphology.

Phenotypic changes of SDF-1^−/−^ hypertrophic chondrocytes of the humeri were mostly observed from E14.5 to E15.5, and the significant difference was lost at E16.5 (**Figure 2**). As we observed the vascularization of the humeri at E15.5 with the expression of SDF-1, we assume that the shortness of the humeri of SDF-1^−/−^ mice is compensated by the effects of circulating factors from blood after neovascularization occurs. To exclude the effect of the vascularization of the humeri, the organ culture of metatarsal bones was performed, and we found that the bone growth was disturbed by lack of SDF-1, and that the discretion was rescued by the administration of SDF-1. These results suggest that SDF-1 is crucial in chondrocyte differentiation at the prevascularized stage, and the functions of SDF-1 in chondrocyte differentiation are totally apart from roles on the recruitment of MSCs nor HSCs.

The bone growth is driven primarily by the rate of production of hypertrophic chondrocytes from proliferating chondrocytes [44]. In the metatarsal explant culture, lack of SDF-1 resulted in the decreased expression of Col X with disturbance of the bone growth (**Figures 6A, D**). This fact suggests SDF-1 signaling is important for the hypertrophic conversion of proliferating chondrocytes. Interestingly, the appearance of the calcified zones was delayed in the explant culture of SDF-1^−/−^ mice (**Figures 5B, C**). Since the hypertrophic conversion is a prerequisite of calcification of chondrocytes, SDF-1 is suggestively crucial on the hypertrophic conversion and the subsequent calcification of chondrocytes. Indeed, the delayed calcification was also found in the SDF-1^−/−^ humeri (**Figure 2A**), and the disturbed hypertrophic conversion led to the shortness of calcified zones as well as that of the hypertrophic zones in SDF-1^−/−^ mouse embryos (**Figure** **2B**). Furthermore, the stimulation of SDF-1 to SDF-1^−/−^ metatarsals increased the expression of Col X mRNA (**Figure 6D**). Since SDF-1 is expressed at prehypertrophic and hypertrophic chondrocytes (**Figure 1**) with CXCR4 expression being the strongest in the hypertophic chondrocytes [**36**], the effects of SDF-1 on chondrocyte differentiation may have occurred mainly in the prehypertrophic and hypertrophic zones as enhancement of chondrocyte hypertrophy.

However, the present study contains a few notable limitations. Firstly this study does not show the intracellular signaling of SDF-1 in chondrocyte differentiation. **Figure 5A** showed that formation of actin stress fibers in untreated wild-type chondrocytes and chondrocytes treated with SDF-1 and TF14016 was similar, but a slight actin stress fiber formation was observed in cells treated with SDF-1 and PTX. Since PTX inactivates Gi, actin stress fiber formation with SDF-1 and PTX may also have resulted from the increase of F-actin and contraction of actomyosin through G12/13. Actin stress fiber formation for the hypertrophic transition by SDF-1 may strongly depend on Gi in addition to G12/13. This result is compatible with a previous report that RhoA/ROCK signaling, downstream of G12/13, inhibited hypertrophic differentiation [43]. SDF-1 signal transduction pathway in chondrocytes is still elusive and remains to be investigated in the future.

Another relevant limitation of this study is that this study did not elucidate the association of SDF-1 signaling with other signaling molecules regulating the hypertrophic conversion of chondrocytes. RhoA/ROCK signaling, the only signaling shown to associate with actin organization during chondrogenesis, suppresses Sox9 expression [24]. Sox9 mRNA expression, however, did not show any significant difference between wild-type and SDF-1^−/−^ metatarsals and primary chondrocytes in monolayer in this study (**Figures 6D, E**). SDF-1 may regulate the hypertrophic conversion in chondrocytic differentiation and the bone growth without the involvement of Sox9, but this assumption requires further investigations.

In addition, the influence of genetic background of SDF-1^−/−^ mice cannot completely be removed. As about half of SDF-1^−/−^ mice start to die after E16.5 for unknown reasons, contamination of the dying embryos can affect the results. These dying embryos are easily distinguishable by their relatively small size and dark red color and we did not use these embryos for the experiments. However, there is still a possibility of the contamination. One of the solutions for this issue is the use of conditional knock-out mice. As there is no report showing mice that express Cre recombinase specifically in SDF-1-expressing prehypertrophic and/or hypertrophic chondrocytes, this issue should be pursued in the near future.

In conclusion, we investigated the role of SDF-1 in chondrocyte differentiation and revealed that SDF-1 stimulates bone growth by mediating chondrocyte hypertrophy and regulates actin polymerization.

## Materials and Methods

### Reagents

Recombinant mouse CXCL12/SDF-1α was purchased from R&D Systems (Minneapolis, MN). Pertussis toxin (PTX) was purchased from Sigma-Aldrich (St. Louis, MO).

### Mice

All animal studies were conducted in accordance with principles by Kyoto University Committee of Animal Resources, based on International Guiding Principles for Biomedical Research Involving Animals. All procedures for this study were approved by Kyoto University Committee of Animal Resources (Permit Number: MedKyo 11260). The generation of SDF-1^−/−^ mice has been previously described [45]. Heterozygotes were maintained and backcrossed more than 10 times with C57BL/6NCrSlc mice. Homozygous mutant embryos were present at the expected Mendelian ratios until E15.5, and about half of the SDF-1^−/−^ embryos were dead at E18.5. The embryos that die within the uteri were slightly small in dark red color, and these were not used for the experiments.

### Mouse Rib Fracture Model

Mouse rib fracture model was created using 6-week-old wild-type (C57BL/6NCrSlc) mice as previously described [46]. The mice were sacrificed at day 10 for histological analysis.

### Histological Analysis and Measurement of the Ratios of Proliferating and Hypertrophic Chondrocytes

Specimens were processed to paraffin-embedded sections with a thickness of 5–7 µm, and stained with hematoxylin, eosin and alcian blue. The areas of proliferating and hypertrophic cartilages were measured by computer tracing as previously described [47]. Immunohistochemical analysis was performed to detect SDF-1 protein as previously described [22].

### Visualization of Cytoskeleton in Hypertrophic Chondrocytes of the Humeri

The humeri of embryonic day 15.5 (E15.5) were isolated from wild-type embryos or crosses of SDF-1^+/−^ mice. Samples were frozen in O.C.T. Compound (Sakura Finetek Japan, Tokyo, Japan). Tissues were cryosectioned at 12 µm, and briefly airdried. Sections were fixed with 4% paraformaldehyde for 20 m, followed by two washes in phosphate-buffered saline (PBS). The membranes were permeabilized with 0.5% Triton X-100/PBS solution for 20 m, and incubated in the dark for 2 h with 100 nM Rhodamine-Phalloidin (Cytoskelton, Denver, CO) and 0.01% SYBR Green I nucleic acid gel stain (Life Technologies, Carlsbad, CA). Images were taken with Nicon P-Eclipse C1 confocal microscopy equipped with objective lens 60X/1.40 oil.

### Isolation and Culture of Primary Chondrocytes

Primary chondrocytes were isolated from embryonic ribs at E15.5 and cultured in Dulbecco’s Modified Eagle Medium, which was regarded as post passage 0 (pp 0). When the cells became pre-confluent, the cells were trypsinized and were passaged (pp 1). Cell suspensions at the cell concentration of 1×10*5 cells/ml were used for actin polymerization assay. Primary chondrocytes (pp 0) were also cultured for 3 weeks in chondrogenic medium supplemented with insulin (10 µg/ml), transferrin (5.5 µg/ml), and sodium selenite (5 ng/ml) (Sigma, St. Louis, MO) to induce chondrocyte differentiation, as previously described [48].

### Actin Polymerization Assay of Primary Chondrocytes

To investigate the function of SDF-1, the cells were treated with SDF-1, TF14016, and/or PTX added to the medium for 1 m, 5 m, 10 m, 30 m, 1 h, 3 h, or 24 h. Then the cells were fixed with 4% paraformaldehyde for 10 m, and stained with 100 nM Rhodamine-Phalloidin and 0.05% 4′,6-diamidino-2-phenylindole for 30 s or 0.01% SYBR Green according to the manufacture’s protocol. Images were taken Olympus IX70 laser microscopy or Nicon P-Eclipse C1 confocal microcopy, and analyzed with Image J as described before [49].

### Metatarsal Organ Culture

Three central metatarsal rudiments were isolated from each hind limb of E15.5 embryos. Every three metatarsals from each limb were placed into each well of a 24-well plate containing 0.5 ml of organ culture medium as previously described [50]. The day of explant harvest was regarded as day 0 and the culture medium was replaced at day 1, and 4. SDF-1 were applied to the well containing the explants from the right hind limb, and the ones from the left hind limb were treated with culture medium alone as controls. Cultures were observed and photographed with an Olympus SZX 12 dissecting microscope at day 1, 3, 5, and 7. The cultured metatarsals were harvested at day 7 for histological procedure, and at day 3 or 7 for RNA extraction.

### RNA Extraction and Quantitative Real-time PCR

Total RNA from cultured metatarsal bones were snap frozen in liquid nitrogen, homogenized, and extracted total RNA using High Pure RNA Tissue Kit (Roche Diagnostics, Penzberg, Germany). Total RNA from primary chondrocyte was extracted using High Pure RNA Isolation Kit (Roche Diagnostics). The RNA was reverse-transcribed and real-time quantitative PCR was performed using FastStart Universal SYBR Green Master (Roche Diagnostics) and ABI7500 (Life Technologies) according to the manufacturer’s instructions. All of gene expression data were normalized against glyceraldehyde-3-phosphate dehydrogenase (GAPDH, forward primer, 5′- AGGTCGGTGTGAACGGATTTG, and reverse primer 5′- TGTAGACCATGTAGTTGAGGTCA). Expression patterns of Col X (forward primer, 5′- AGGCTACCTGGATCAGGCTTC, and reverse primer 5′-ACATTCTTTTCAGCCTACCTCC), and Sox9 mRNA (forward primer, 5′-GAGCCGGATCTGAAGAGGGA, and reverse primer 5′- GCTTGACGTGTGGCTTGTTC) were also analyzed. Standard curve was generated by serially diluted plasmids containing PCR amplicon sequences. Plasmids were synthesized using pTAC-1 vector (BioDynamics Laboratory, Tokyo, Japan).

### Proliferation Assay

BrdU incorporation was assessed by the cell proliferation ELISA Biotrack kit (Amersham Biosciences, Piscataway, NJ). BrdU was administrated into the mice at E15.5 2 h before the harvest. BrdU-positive cells were detected immunohistochemically as previously described [22].

### Statistical Analysis

Data were analyzed with Student’s t test. A p value less than 0.05 was considered statistically significant.

## Supporting Information

Figure S1 **BRd-U staining of embryonic humeri.** **A**: BRd-U staining of embryonic humeri of wild-type (WT) and SDF-1^−/−^ (KO) mice. Embryonic humeri of wild-type and SDF-1^−/−^ mice were processed to paraffin-embedded sections and stained with Brd-U. B: The percentage of positive cells for BRd-U was calculated in WT and KO humeri.
(TIF)
