# Supplementary figures and images for: Stromal Cell-Derived Factor 1 Regulates the Actin Organization of Chondrocytes and Chondrocyte Hypertrophy
Source: PLoS One. 2012 May 18;7(5):e37163. doi: 10.1371/journal.pone.0037163 (PMC3356379; doi:10.1371/journal.pone.0037163)

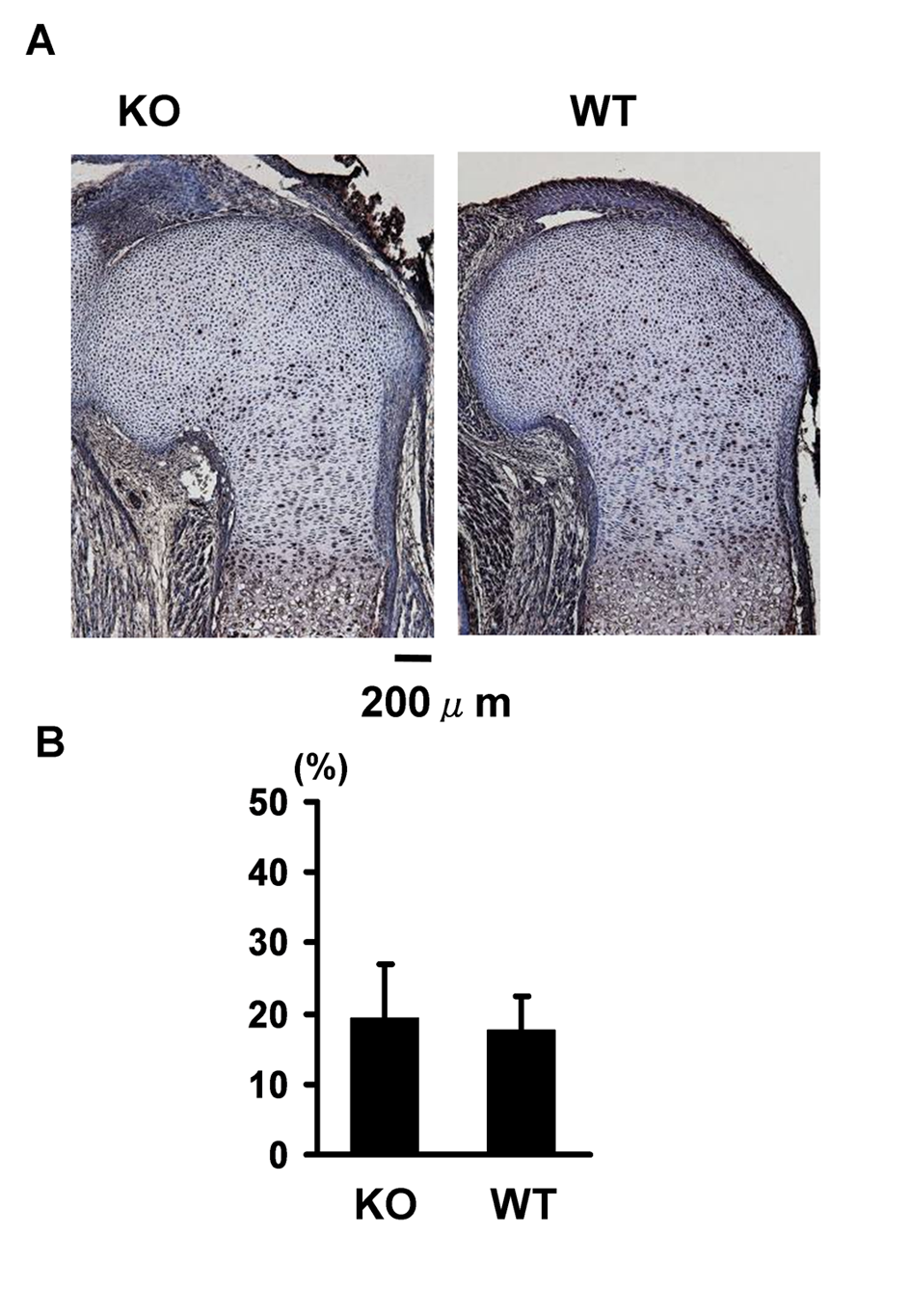

Supplement: Figure S1 — BRd-U staining of embryonic humeri. A: BRd-U staining of embryonic humeri of wild-type (WT) and SDF-1−/− (KO) mice. Embryonic humeri of wild-type and SDF-1−/− mice were processed to paraffin-embedded sections and stained with Brd-U. B: The percentage of positive cells for BRd-U was calculated in WT and KO humeri. (TIF) [file pone.0037163.s001.tif]
